# *Slc26a2*-mediated sulfate metabolism is important in tooth development

**DOI:** 10.1242/dmm.052107

**Published:** 2024-12-10

**Authors:** Yuka Yoshida, Toshihiro Inubushi, Mika Yokoyama, Priyanka Nag, Jun-ichi Sasaki, Ayaka Oka, Tomoya Murotani, Renshiro Kani, Yuki Shiraishi, Hiroshi Kurosaka, Yoshifumi Takahata, Riko Nishimura, Satoshi Imazato, Petros Papagerakis, Takashi Yamashiro

**Affiliations:** ^1^ Department of Orthodontics and Dentofacial Orthopedics, Osaka University Graduate School of Dentistry, Osaka 565-0871, Japan; ^2^ Department of Molecular and Cellular Biochemistry, Osaka University Graduate School of Dentistry, Osaka 565-0871, Japan; ^3^ Genome Editing Research and Development Unit, Osaka University Graduate School of Dentistry, Osaka 565-0871, Japan; ^4^ Department of Dental Biomaterials, Osaka University Graduate School of Dentistry, Osaka 565-0871, Japan; ^5^ Division of Biomedical Engineering, University of Saskatchewan, Saskatoon, SK S7N 5E5, Canada; ^6^ College of Dentistry, University of Saskatchewan, Saskatoon, SK S7N 5E5, Canada

**Keywords:** Tooth development, Odontoblasts, Extracellular matrix, Sulfate metabolism, Matrix biology, SLC26A2

## Abstract

The sulfate transporter gene *SLC26A2* is crucial for skeletal formation, as evidenced by its role in diastrophic dysplasia, a type of skeletal dysplasia in humans. Although SLC26A2-related chondrodysplasia also affects craniofacial and tooth development, its specific role in these processes remains unclear. In this study, we explored the pivotal roles of SLC26A2-mediated sulfate metabolism during tooth development. We found that *Slc26a2* was predominantly expressed in dental tissues, including odontoblasts and ameloblasts. *Slc26a2* knockout (*Slc26a2-KO-Δexon2*) mice exhibited distinct craniofacial abnormalities, such as a retrognathic upper jaw, small upper incisors and upper molar hypoplasia. These mice also showed flattened odontoblasts and loss of nuclear polarity in upper incisors and molars, with significant reductions in odontoblast differentiation markers *Dspp* and *Dmp1*. *Ex vivo* and *in vitro* studies further revealed dentin matrix hypoplasia, tooth root shortening and downregulation of Wnt signaling in *Slc26a2*-deficient cells. These findings highlight the crucial role of SLC26A2-mediated sulfate metabolism in tooth development and offer insights into the mechanisms underlying dental abnormalities in patients with SLC26A2-related chondrodysplasias.

## Teaser

**Summary:** *Slc26a2*-mediated sulfate metabolism plays a critical role in tooth development, with deficiencies leading to disrupted odontoblast differentiation and downregulation of Wnt signaling.

## INTRODUCTION

Sulfate [or sulfate ion (SO_4_^2−^)] is an anion involved in a wide range of significant biological processes, including biosynthesis and metabolism of a variety of endogenous biological molecules, and is important for cell growth and development of the organism (Foster and Mueller, 2018; Soares da Costa et al., 2017). Because sulfate cannot freely pass through the plasma membranes of cells, transport mechanisms are required for the movement of sulfate into and out of mammalian cells (Alper and Sharma, 2013). Such mechanisms are also necessary for sulfate absorption from the gastrointestinal tract and re-absorption by the renal tubules (Seidler and Nikolovska, 2019). After sulfate is transported into the cytoplasm or nucleus, 3'-phosphoadenosine 5'-phosphosulfate (PAPS) is synthesized from ATP and sulfate by two enzymatic reactions – catalyzed by ATP sulfurylase and adenosine 5'-phosphosulfate kinase. After PAPS is transported to the Golgi apparatus, sulfotransferases transfer sulfate groups from PAPS to glycosaminoglycans and tyrosine. In addition, cytosolic sulfotransferases transfer sulfate groups from PAPS to steroid hormones in cytosol (Klaassen and Boles, 1997). However, part of the sulfate supply is also known to be derived from breakdown of the sulfur-containing amino acids cysteine and methionine (Elgavish and Meezan, 1991; Markovich and Aronson, 2007).

In mammals, the Slc26 gene family, which encodes anion transporters, consists of 11 genes – *Slc26a1-11*. The Slc26 gene family encodes transporters of a broad range of anionic substrates, including sulfate, HCO^3−^, Cl^−^, oxalate, I^−^ and formate. *Slc26a1* and *Slc26a2* encode the proteins SAT1 and DTDST, respectively, which are sulfate/chloride exchangers that function as cell membrane sulfate transporters and enable the intracellular transport of inorganic sulfate (Kere, 2006). In humans, variants in the *SLC26A2* gene cause a spectrum of recessively inherited chondrodysplasias. Although the phenotype differs according to the type of *SLC26A2* variants, the main clinical features are short stature, joint contractures, club feet, shortening of the limbs and a waddling gait (Cai et al., 2015). More recent reports also suggest that *SLC26A2* variants are associated with a wide range of clinical manifestations in the craniomaxillofacial region, including large upper facial height, micrognathia, high palate, cleft palate (25-60%), tooth agenesis (30%) and microdontia (Härkönen et al., 2021). Variants in the other eight members of the SLC26 family have also been implicated in human disease. However, unlike *SLC26A2* variants, variants in the other SLC26 family members do not induce any abnormalities in the skeletal system or craniofacial region.

The function of *Slc26a2* in mammals has been investigated using genetic mouse models. Forlino et al. (2005) generated a DTDST knock-in mouse model harboring human variants, and the mice showed partial loss of function of the sulfate transporter. These mice exhibited a short stature, joint contracture, reduced Toluidine Blue staining of cartilage and irregular chondrocyte size (Forlino et al., 2005). Zheng et al. (2019) generated *Slc26a2*^−/−^ mice to investigate the effects of SLC26A2 deficiency on chondrodysplasia. Although patients with variants in the *SLC26A2* gene reportedly show abnormalities in the craniomaxillofacial region, including dwarf teeth and congenital absence of teeth (Karlstedt et al., 1996), the role of SLC26A2 during tooth development has not been fully elucidated.

In the present study, we investigated the pattern of *Slc26a2* expression in developing tooth germs, performed morphological and histological evaluations of tooth germs in *Slc26a2* knockout mice, and examined the effects of *Slc26a2* deficiency on enamel and dentin matrix formation using kidney-capsule grafting. This is the first study to demonstrate the pivotal role of the SLC26A2-mediated sulfate transporter during tooth development.

## RESULTS

### *Slc26a2* is predominantly expressed among sulfate transporter family genes during tooth development

**Fig. 1.**
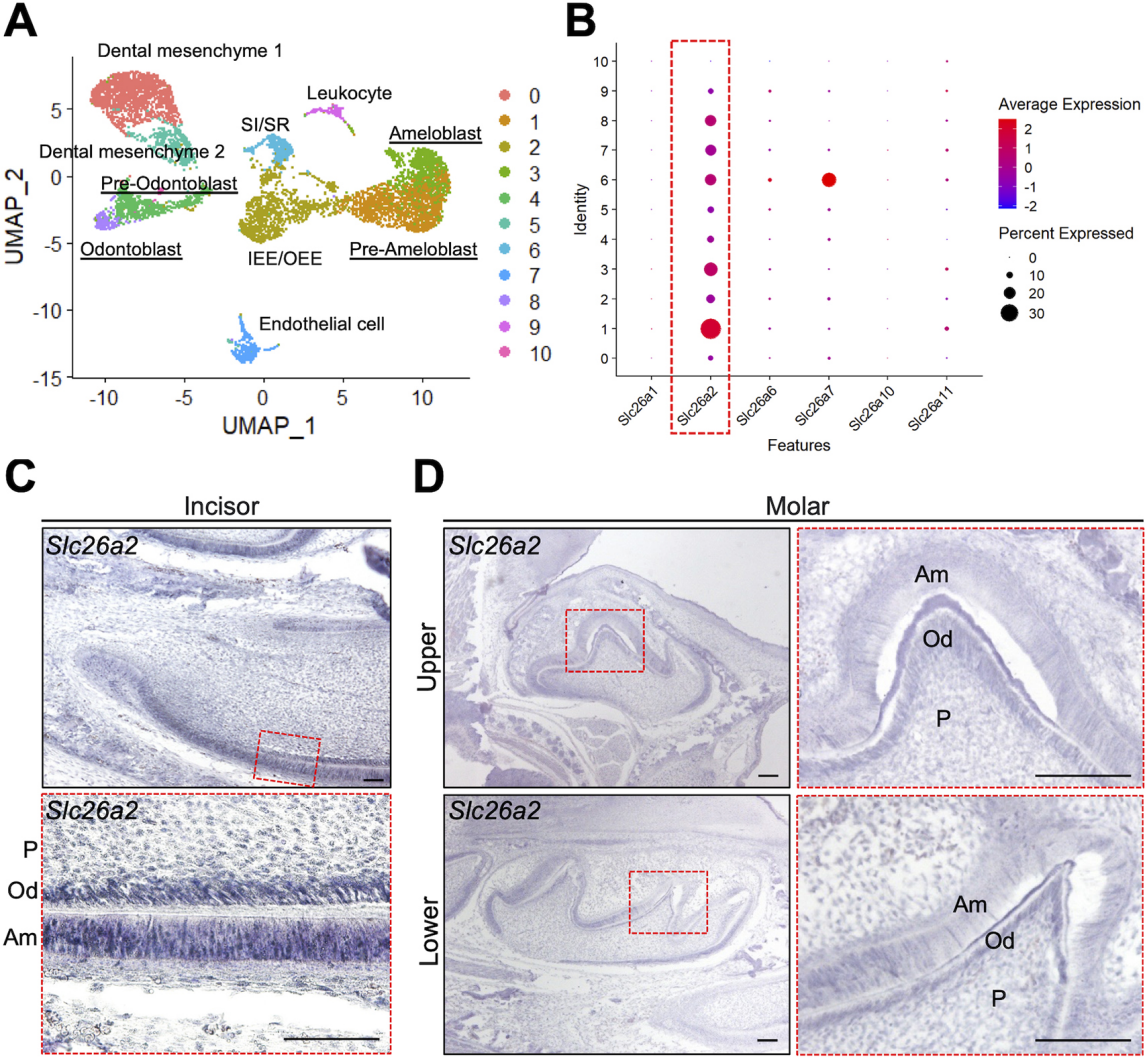
**Pattern of *Slc26a2* expression during tooth development in mice.** (A) Re-analysis of a public single-cell RNA-sequencing dataset on isolated mouse incisors at postnatal day (P)0. Ten cell population clusters, including odontoblasts, pre-odontoblasts, ameloblasts and pre-ameloblasts, are identified in mice tooth germs at P0. IEE, inner enamel epithelium; OEE, outer enamel epithelium; SI, stratum intermedium; SR, stellate reticulum; UMAP, uniform manifold approximation and projection. (B) Dot plot showing the expression of Slc26 family members in various clusters. Dot size represents the percentage of cells expressing a specific gene. The intensity of color indicates the average expression level for a gene in the cluster. *Slc26a2* is predominantly expressed in mouse tooth germs. (C,D) The expression of *Slc26a2* during tooth development at embryonic day (E)18.5. Frontal sections through the upper (D, top row) and lower (D, bottom row) molar, and sagittal sections through the lower incisor (C), in wild type, processed by RNA *in situ* hybridization. Dashed red line boxes demarcate the magnified areas. Am, ameloblast; Od, odontoblast; P, dental pulp. Scale bars: 100 μm. All samples are wild-type C57B6/J.

To understand the role of SLC26A2 in tooth development, we quantitatively analyzed the expression of sulfate transporter family genes, including *Slc26a1* and *Slc26a2*, in developing tooth germ. We re-analyzed a public single-cell RNA sequencing dataset on isolated mice incisors at postnatal day (P)0. We identified 11 cell population clusters of odontoblasts, sub-odontoblasts, dental mesenchyme 1 and 2, ameloblasts, pre-ameloblasts, inner enamel epithelium or outer enamel epithelium, stratum intermedium or stellate reticulum, leukocytes, erythrocytes and endothelial cells (Fig. 1A; Fig. S1). We next characterized the expression of *Slc26a1*, *Slc26a2*, *Slc26a6*, *Slc26a7*, *Slc26a10* and *Slc26a11* in the dataset. A dot plot showed that *Slc26a2* was more strongly expressed in all clusters, including ameloblast and odontoblast clusters, than other sulfate transporter family genes (Fig. 1B).

We next used *in situ* hybridization to confirm the expression pattern of *Slc26a2* in tooth development in mice at embryonic day (E)18.5. The prominent expression of *Slc26a2* was observed in odontoblasts and ameloblasts at E18.5 (Fig. 1C). Interestingly, at each developmental stage, the expression of *Slc26a2* was found to be markedly higher than that of *Slc26a1* (Fig. S2A)*.* Furthermore, the expression of *Slc26a2* increased with developmental stage, with craniofacial formation beginning at E9.5 and the tooth bud forming at E13.5 (Fig. S2A). At E18.5, the expression of *Slc26a2* was higher than that of *Slc26a1* in the upper molars (Fig. S2B). These results suggest that *Slc26a2* plays a major role in the transport of sulfate ions through the cell membrane during tooth development.

### *Slc26a2*-deficient mice show a hypoplastic maxilla and hypoplasia of the upper teeth

**Fig. 2.**
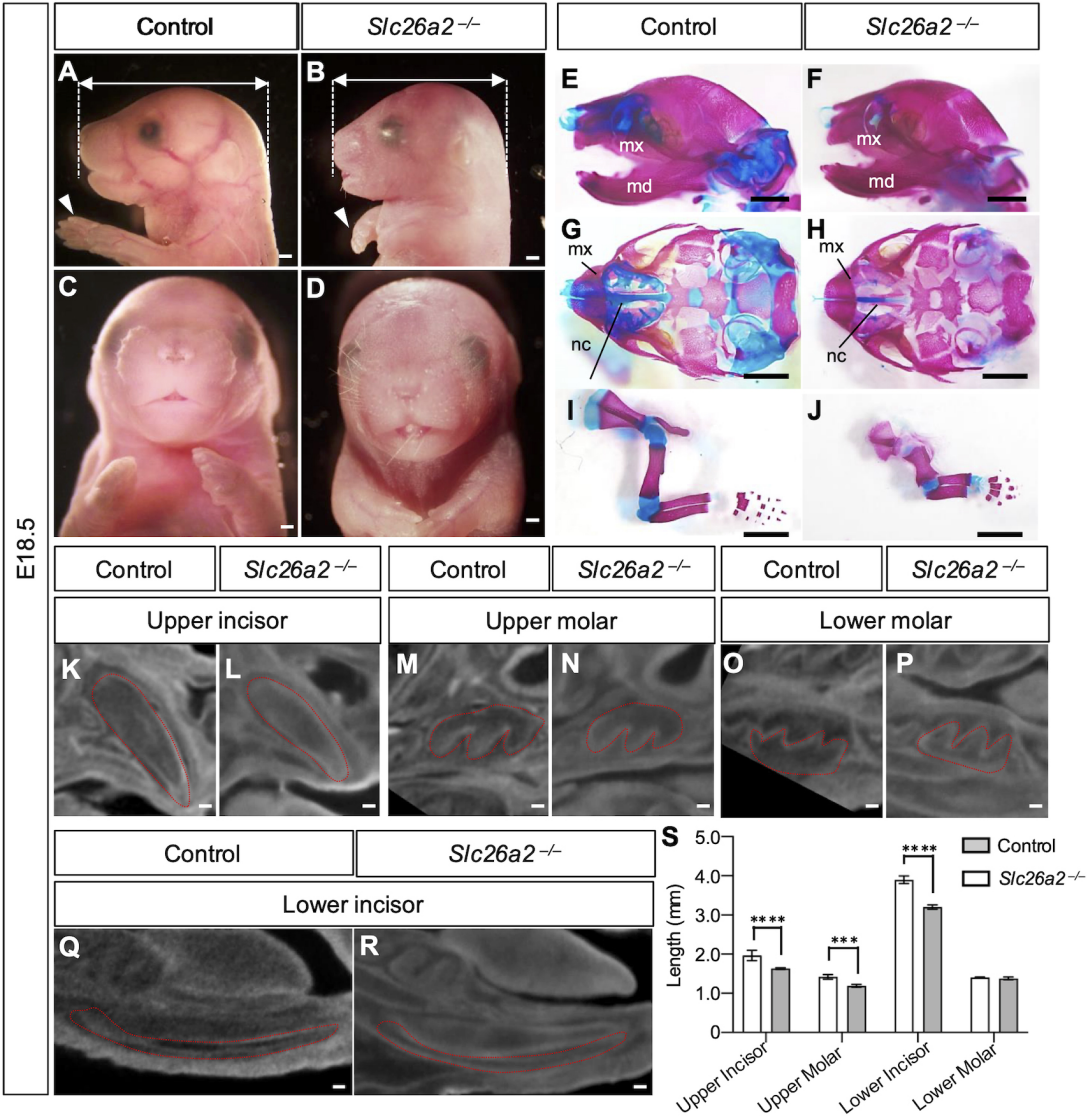
***Slc26a2-KO-Δexon2* mice show skeletal and dental abnormalities.** (A-D) Gross phenotype of *Slc26a2-KO-Δexon2* mice at E18.5. Whole-mount images of *Slc26a2-KO-Δexon2* mice revealed hypoplasia of the maxilla and very short limbs (white arrowheads). Lateral (A,B) and frontal (C,D) views of the head are shown. Scale bars: 1 mm (A,B), 500 μm (C,D). (E-J) Whole-mount skeletal preparations of craniofacial bones (E,F), cranial base (G,H) and limbs (I,J) in control and *Slc26a2-KO-Δexon2* mice at E18.5. Whole-mount skeletal preparations show that *Slc26a2-KO-Δexon2* mice had chondrodysplasia and reduced Alcian Blue staining of the limbs and cranial cartilage. md, mandible; mx, maxilla; nc, nasal cavity. Scale bars: 2 mm. (K-R) Contrast-enhanced micro-computed tomography (CT) images of control and *Slc26a2-KO-Δexon2* mouse tooth germs at E18.5. The upper molar and incisor widths of *Slc26a2-KO-Δexon2* mice are smaller than those of the control. Scale bars: 50 μm. (S) Quantitative assessment of upper and lower tooth size in control and *Slc26a2-KO-Δexon2* mice at E18.5. *Slc26a2-KO-Δexon2* mice show hypoplasia of the upper and lower incisors and upper molars, but not of the lower molars, compared to those of the control mice. *n*=3; ***P<0.001, ****P<0.0001 (two-way ANOVA).

To investigate the role of *Slc26a2* deficiency in tooth development, we generated *Slc26a2* knockout mice by targeted deletion of *Slc26a2* exon 2 (*Slc26a2-KO-Δexon2*) using the CRISPR-Cas9 gene-editing method (Makino et al., 2016). Previous reports showed that *Slc26a2* knockout (KO) mice died immediately after birth, with no respiratory movement and an overall skeletal phenotype characterized by a short neck, small chest and very short limbs (Zheng et al., 2019). Consistently, *Slc26a2-KO-Δexon2* mice died immediately after birth owing to respiratory abnormalities. Whole-mount images of *Slc26a2-KO-Δexon2* mice at E18.5 revealed hypoplasia of the maxilla rather than the mandible (Fig. 2A-D; Fig. S3). Micro-computed tomography (CT) of *Slc26a2-KO-Δexon2* mice demonstrated a short stature, small chest and very short limbs. The long tubular bone was shorter in length and longer in diameter than that of the control mice (Fig. S4A,B). Whole-mount skeletal preparations demonstrated chondrodysplasia and reduced Alcian Blue staining of the cartilage in *Slc26a2-KO-Δexon2* mice (Fig. S4C,D and Fig. S5). In the cranio-maxillofacial region, *Slc26a2-KO-Δexon2* mice showed a hypoplastic maxilla, hypoplasia of nasal cartilage (Fig. 2E,F), small cranial base (Fig. 2G,H) and short ribs (Fig. 2I,J), while the mandible size remained consistent with that of control mice (Fig. S5). Additionally, we observed reduced Alcian Blue staining intensity in the nasal septum, synchondroses of the cranial base, costal cartilage and growth plate cartilage at E18.5 (Fig. 2; Fig. S5). Furthermore, at E15, Meckel's cartilage showed decreased Alcian Blue staining intensity and hypoplasia, indicating impaired cartilage formation in these regions (Fig. S6). At E18.5, mutant and control tooth germs were examined by contrast-enhanced micro-CT (Fig. 2K-R). *Slc26a2-KO-Δexon2* mice showed a shorter anterior–posterior length of the upper and lower incisor, measured from the cervical loop to the incisor tip, compared to that of control mice. Similarly, the upper molar tooth germs in *Slc26a2-KO-Δexon2* mice were shorter with regard to both crown proximal–central width and height compared to those of control mice. In contrast, there were no significant differences in the width or height of the lower molars between *Slc26a2-KO-Δexon2* and control mice (Fig. 2S).

### *Slc26a2* deficiency leads to impaired differentiation of odontoblasts and ameloblasts

**Fig. 3.**
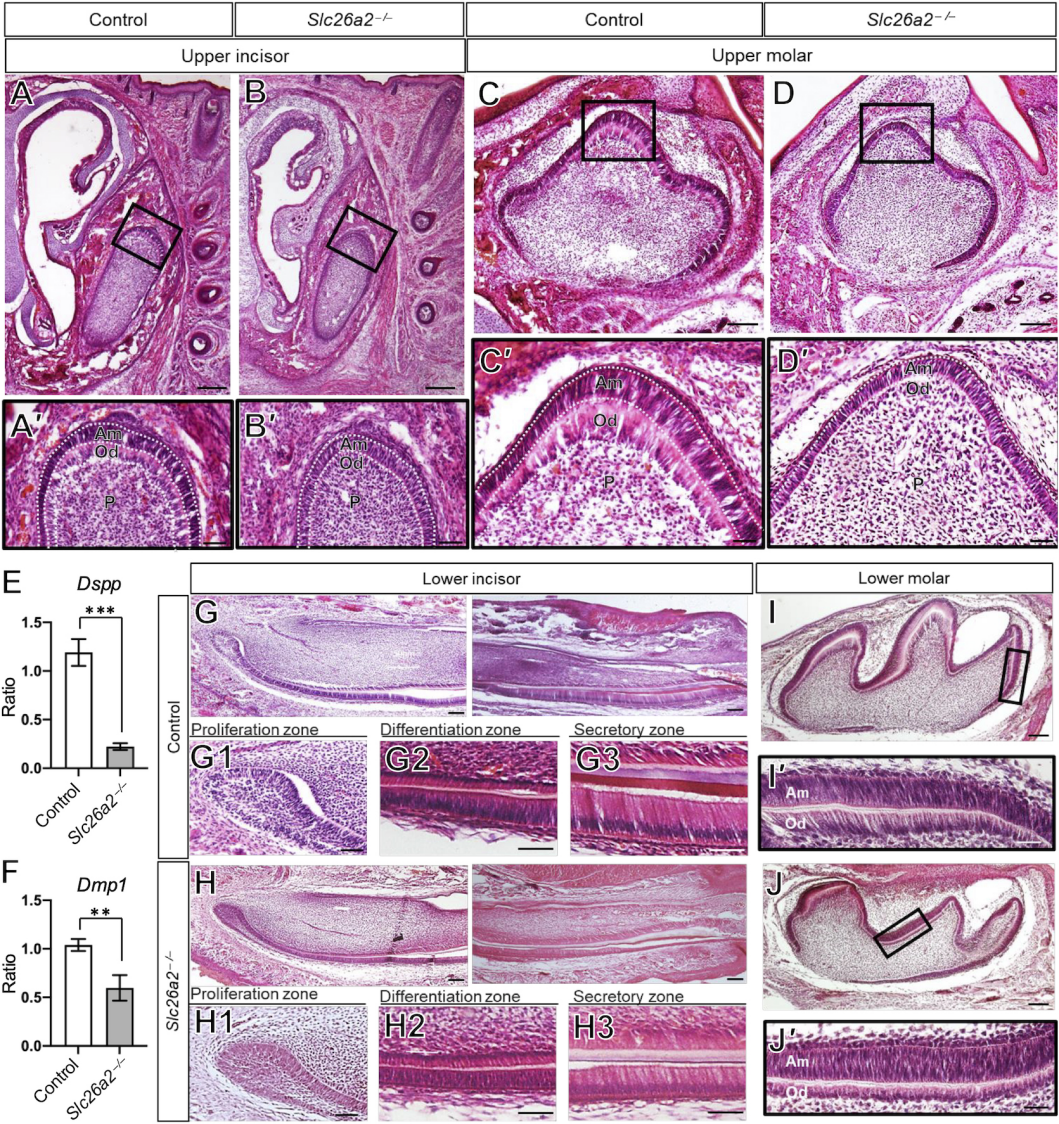
***Slc26a2* deficiency leads to impaired differentiation of odontoblasts in the upper tooth.** (A,B,C,D) Hematoxylin and Eosin (H&E) staining of frontal sections of upper incisor and molar tooth germ. Upper incisor and upper molar tooth germ in *Slc26a2-KO-Δexon2* mice show flattened odontoblasts cell body and nuclei, and loss of intracellular polarity. (A′,B′,C′,D′) Magnified views of the boxed areas in A, B, C and D, respectively. (E,F) Quantitative PCR (qPCR) analysis of *Dspp* and *Dmp1* expression in odontoblasts of upper tooth germ at E18.5. In *Slc26a2-KO-Δexon2* mice, the expression of *Dspp* and *Dmp1* in upper molar tooth germ was significantly decreased compared to that in control mice. *n*=3; **P<0.01, ***P<0.001 (unpaired two-tailed Student's *t*-test). (G-J′) H&E staining of sagittal sections of lower incisor (G-H3) and lower molar (I-J′) tooth germ. The overall structure of lower molar tooth germ is comparable in control and *Slc26a2-KO-Δexon2* mice (I,J). In the high-magnification images of the differentiation zone (G2,H2), the pre-secretary ameloblast layer is larger in *Slc26a2-KO-Δexon2* mice than that in control mice. The heights of secretary and mature ameloblasts are lower in *Slc26a2-KO-Δexon2* mice than those in control mice (G3,H3). Reduction in enamel and dentin thickness is observed in the incisor tooth germ in *Slc26a2-KO-Δexon2* mice (G3,H3). The tooth phenotype in lower molar tooth germ in *Slc26a2-KO-Δexon2* mice is much milder than that in upper molar tooth germ (I-J′). Am, ameloblast; Od, odontoblasts; P, dental pulp. Scale bars: 200 μm (A,B); 100 μm (C,D,G,H,I,J); 50 μm (A′,B′,C′,D′,G2,G3,H2,H3,I′,J′); 20 μm (G1,H1).

We performed a histological evaluation to clarify the effects of *Slc26a2* deficiency on tooth development. Upper incisors and molars in *Slc26a2-KO-Δexon2* mice had flattened odontoblasts and nuclei that did not show intracellular polarity (Fig. 3A-D′). The height of pre-secretory ameloblasts was lower in *Slc26a2-KO-Δexon2* mice than in control mice.

To further examine the effect of *Slc26a2* deficiency on odontoblast differentiation, we evaluated the expression of the odontoblast marker genes dentin sialoprotein (*Dspp*) and dentin matrix protein 1 (*Dmp1*), which encode non-collagenous organic substances in dentin and show increased expression in association with odontoblast differentiation (Chen et al., 2016; Yamashiro et al., 2007). In *Slc26a2-KO-Δexon2* mouse tooth germ, the expression of *Dspp* and *Dmp1* in upper molars was significantly decreased compared to that in control tooth germ (Fig. 3E,F). The tooth phenotype in *Slc26a2-KO-Δexon2* mice was much milder in lower molar tooth germs than that in upper molar tooth germs (Fig. 3G-J′). Taken together, these results demonstrate that *Slc26a2* is required for odontoblast differentiation of upper molars in mice.

### *SLC26A2*-deficient human dental pulp stem cells show defective differentiation into odontoblasts

**Fig. 4.**
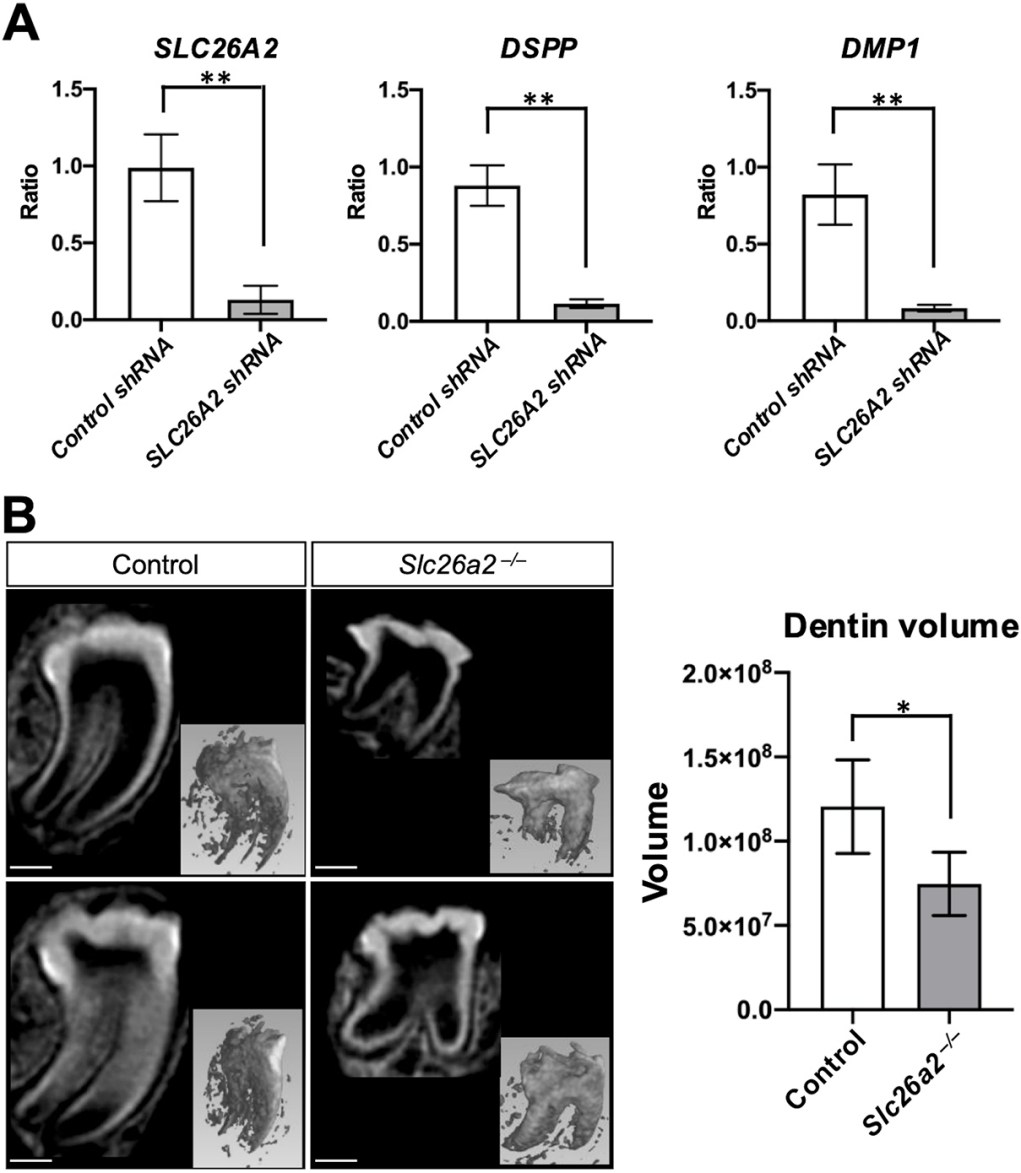
**Analysis of the effect of *Slc26a2* deletion on dentinogenesis.** (A) *SLC26A2*-depleted and control human dental pulp stem cells (hDPSCs) were cultured for 7 days in odontoblast differentiation medium. The expression of *SLC26A2*, *DSPP* and *DMP1* was evaluated by qPCR. Note that *SLC26A2*, *DSPP* and *DMP1* expression was significantly decreased in *SLC26A2*-depleted cells compared to that in the control cells. *GAPDH* was used as an internal control for normalization. Means±s.d. (*n*=3) are shown. **P<0.01 (two-way ANOVA). (B) *Ex vivo* organ culture of tooth germ by implantation of the tooth germ under the kidney capsule of nude mice. After 4 weeks of organ culture, the implanted tooth germs were collected and analyzed by micro-CT. Sagittal sections of control and *Slc26a2-KO-Δexon2* mice tooth germ micro-CT images show hypoplasia of the tooth crown, consisting of enamel and dentin. Tooth root shortening was also observed in *Slc26a2-*deficient tooth germ. Scale bars: 450 μm. (C) Quantitative assessment of the dentin volume demonstrated a significant reduction in *Slc26a2-KO-Δexon2* mouse tooth germ compared to that in control tooth germ. *n*=5; *P<0.05 (two-way ANOVA).

To gain further insight into the effect of *SLC26A2* knockdown on the differentiation of dental pulp stem cells into odontoblasts, human dental pulp stem cells (hDPSCs) were used to analyze the odontoblast differentiation potential. *SLC26A2* knockdown cells were generated via lentivirus-mediated delivery of shRNA, and the expression of *SLC26A2* was decreased by more than 80% in sh-*SLC26A2* knockdown cells (Fig. 4A). In sh-*SLC26A2* knockdown cells, the expression of *DSPP* and *DMP1* was significantly decreased compared to that in control cells (Fig. 4A). This result indicates that *SLC26A2* knockdown can directly affect the differentiation of pulp stem cells into odontoblasts.

### *Slc26a2*-deficient tooth germs show significantly reduced dentin formation compared to control tooth germs in *ex vivo* organ culture under the kidney capsule

Owing to the neonatal lethality of *Slc26a2-KO-Δexon2* mice, it was not possible to assess the effects of *Slc26a2* deficiency on dentin and enamel matrix production in *Slc26a2-KO-Δexon2* mice. We performed *ex vivo* organ culture of tooth germs by implanting the tooth germs under the kidney capsule of nude mice. After 4 weeks of organ culture, the implanted tooth germs were collected and analyzed by micro-CT. The sagittal sections of the micro-CT images showed hypoplasia of the tooth crown, consisting of enamel and dentin (Fig. 4B). A quantitative assessment of the dentin volume demonstrated a significant reduction in *Slc26a2-KO-Δexon2* mouse tooth germ compared to control tooth germ (Fig. 4B). In addition, tooth root shortening was observed in *Slc26a2-*deficient tooth germs (Fig. 4B). These results support our hypothesis that *Slc26a2* deficiency leads to impairment of odontoblast differentiation.

### *Slc26a2* deficiency leads to impaired Wnt signaling in mouse dental papilla mesenchymal cells

**Fig. 5.**
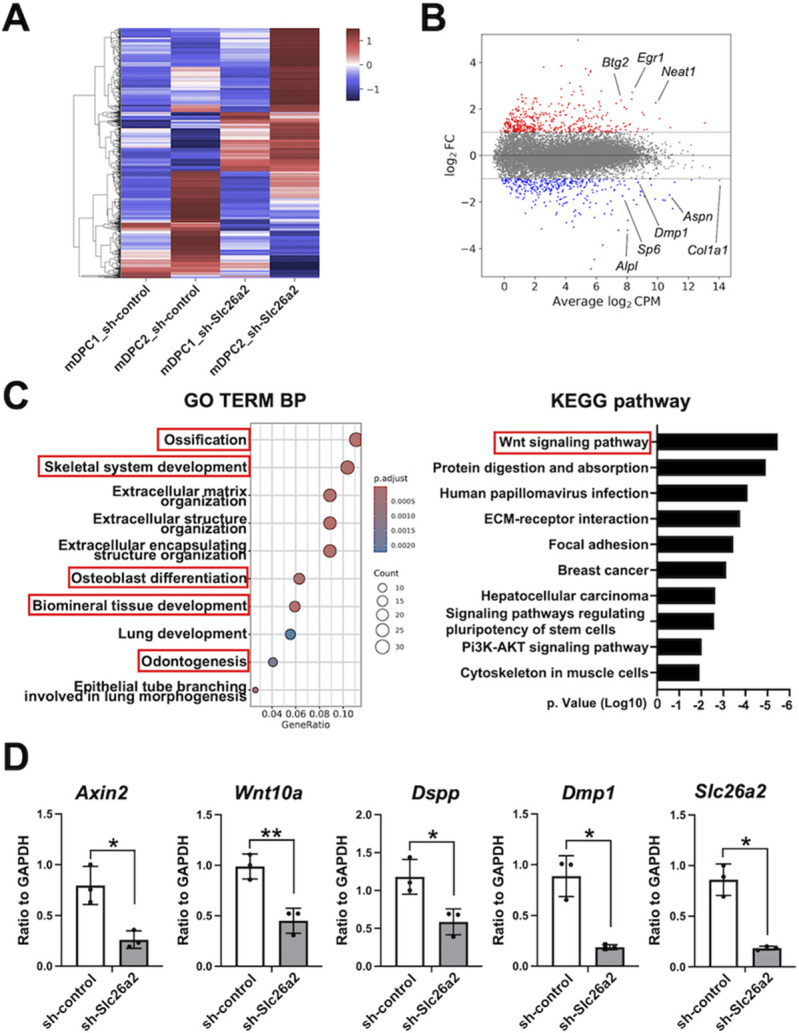
***Slc26a2* deficiency leads to impaired Wnt signaling in mouse dental papilla mesenchymal cells.** (A) Hierarchical clustering heatmap displaying standardized gene expression values ranging from −1.0 to 1.0, with a mean of 0 for sh-*Slc26a2* and sh-control mouse dental papilla mesenchymal cells (mDPCs). Red represents genes with high expression levels; blue indicates genes with low expression levels. (B) Volcano plot visualization of genes upregulated or downregulated in *Slc26a2*-silenced mDPCs. Gene values with |log2FC|>1 were considered differentially expressed genes (DEGs). Red dots represent upregulated genes; blue dots represent downregulated genes; gray dots represent nonsignificantly DEGs (non-DEGs). CPM, counts per million; FC, fold change. (C) Gene Ontology (GO) and Kyoto Encyclopedia of Genes and Genomes (KEGG) enrichment analysis of DEGs. BP, biological process. (D) Expression of *Axin2*, *Wnt10a*, *Dmp1*, *Dspp* and *Slc26a2* in sh-*Slc26a2* and sh-control mDPCs after 3 days of differentiation. *n*=3; *P<0.05, **P<0.01 (unpaired two-tailed Student's *t*-test).

To further examine the molecular mechanisms underlying defective odontoblast differentiation in *Slc26a2*-deficient mice, we performed RNA-sequencing (RNA-seq) analysis of genes exhibiting differential expression in *Slc26a2* knockdown primary mouse dental papilla mesenchymal cells (mDPCs). The heatmap generated through hierarchical clustering displayed distinct gene expression patterns between *Slc26a2-*silenced mDPCs and control mDPCs (Fig. 5A). Additionally, volcano plot analysis highlighted significantly upregulated and downregulated genes [|log2fold change (FC)|>2] in *Slc26a2*-silenced mDPCs (Fig. 5B). Gene Ontology (GO) analysis indicated that several biological processes, such as Ossification, Skeletal system development, Osteoblast differentiation, Biomineral tissue development and Odontogenesis, were downregulated in *Slc26a2-*silenced mDPCs compared to control mDPCs (Fig. 5C). Notably, odontogenesis-related genes – including *Col1a1*, *Aspn*, *Dmp1*, *Axin2*, *Wnt10a*, *Sp6*, *Sp7* and *Fgfr2* – were downregulated in *Slc26a2*-defecient mDPCs compared to control mDPCs. Kyoto Encyclopedia of Genes and Genomes (KEGG) enrichment analysis revealed that several downregulated genes were involved in the Wnt signaling pathway, which was identified as the most significantly enriched pathway (Fig. 5C; Fig. S7). The expression of genes specifically related to Wnt signaling (*Axin2* and *Wnt10a*) and odontogenesis (*Dmp1* and *Dspp*) is presented in Fig. 5D.

### Sulfate transporter defect due to *Slc26a2* deficiency is partly compensated in lower tooth germ

**Fig. 6.**
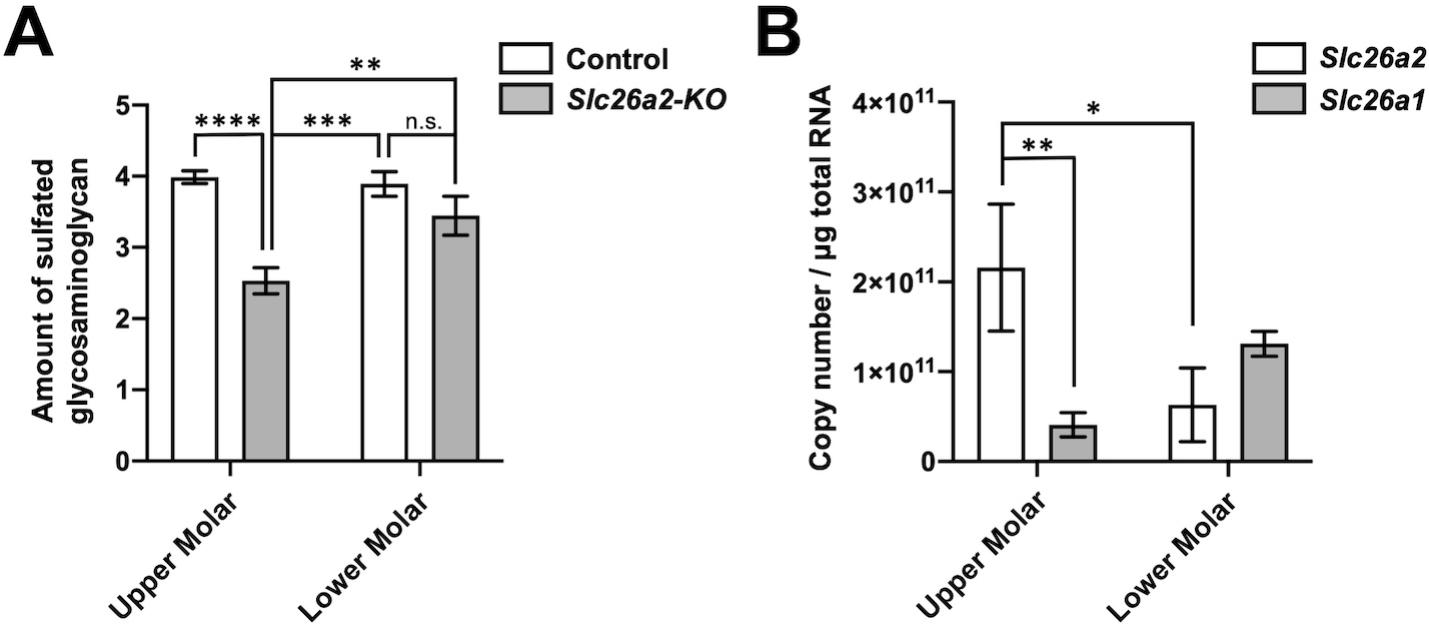
**The sulfate transporter defect resulting from *Slc26a2* deficiency is partly compensated in lower tooth germ.** (A) The total amount of sulfated glycosaminoglycans in upper molar tooth germs was significantly decreased by *Slc26a2* deficiency. *n*=3; n.s., not significant; **P<0.01, ***P<0.001, ****P<0.0001 (two-way ANOVA). (B) The odontoblast layer was extracted by micro-dissection from the sagittal section of upper and lower molars. Absolute quantification was performed to evaluate *Slc26a1* and *Slc26a2* expression. The expression of *Slc26a2* was significantly higher than that of *Slc26a1* in odontoblast of upper tooth germs. Conversely, the expression of *Slc26a1* was higher than that of *Slc26a2* in odontoblasts of lower tooth germs. *n*=3; *P<0.05, **P<0.01 (two-way ANOVA).

Because the phenotype of upper tooth germ is more pronounced than that of lower tooth germ in *Slc26a2-KO-Δexon2* mice (Fig. 3), we hypothesized that the function of *Slc26a2* as a sulfate transporter would be compensated for in the lower tooth germ. To examine the sulfate uptake in the upper and lower tooth germs in *Slc26a2*-deficient and control mice, we quantified the amount of sulfated glycosaminoglycan (GAG), which reflects the sulfate uptake through its transporter. The total amount of sulfated GAG in the upper molar tooth germs was significantly (*P*<0.0001) decreased by *Slc26a2* deficiency (Fig. 6A). In contrast, there was no significant difference in the total amount of sulfated GAG in the lower molar tooth germs between the *Slc26a2*-deficient and control mice (Fig. 6A). Although there was no significant difference in the total amount of sulfated GAG between the upper and lower molar tooth germs in the control group, the total amount of sulfated GAG was significantly decreased in the upper molars compared to that in the lower molars in the *Slc26a2*-deficient group*.*

To assess the genetic redundancy of *Slc26a1*, a homolog of *Slc26a2*, and *Slc26a2*, we performed micro-dissection, which enables the extraction of RNA from odontoblasts with high purity and the absolute quantification of mRNA. As a result, we found that the expression of *Slc26a2* was significantly higher than that of *Slc26a1* in odontoblasts from upper tooth germ (Fig. 6B). Conversely, the expression of *Slc26a1* was higher than that of *Slc26a2* in odontoblasts from lower tooth germ (Fig. 6B). These results suggest that *Slc26a1* is predominantly expressed in lower tooth germs and may compensate for the function of *Slc26a2* in *Slc26a2*-deficient lower tooth germs.

## DISCUSSION

In the present study, we demonstrated that *Slc26a2* is predominantly expressed in dental tissues, with particularly high expression in odontoblasts and ameloblasts, during tooth development (Fig. 1). Deficiency in *Slc26a2* in chondrocytes reportedly disrupts cartilage growth via the attenuation of chondrocyte proliferation and induction of cell death (Zheng et al., 2019). We also confirmed that cell proliferation was decreased, and apoptosis was increased, in *Slc26a2-*deficient chondrocytes *in vivo* (Fig. S8). However, cell proliferation and apoptosis were not significantly altered in the tooth germ of *Slc26a2-KO-Δexon2* mice compared to that of control mice (Fig. S9). The sulfation level was markedly higher in cartilage than in the developing tooth germ (Fig. S10). This indicated that the consumption of the sulfate anion in developing tooth germ is much lower than that in cartilage, and susceptibility to *Slc26a2* deficiency may be dependent on the tissue/cell-specific requirement of sulfate transportation. Interestingly, the tooth phenotype in *Slc26a2-KO-Δexon2* mice was more prominent in the upper incisors and upper molars than in the lower molars (Fig. 3). We found that the expression of *Slc26a1* was higher in the lower tooth than in the upper tooth (Fig. 6B), suggesting that SLC26A1 is able to compensate for SLC26A2 in lower tooth germ. In fact, *Slc26a2* deficiency did not affect the amount of sulfated GAG in the lower tooth germ. However, a significant difference was observed in the upper tooth germ of *Slc26a2-KO-Δexon2* mice compared to control mice (Fig. 6A). The upper and lower jaws are derived from the first branchial arches. Dlx transcriptional factors are regionally expressed within branchial arches and are implicated in regulating jaw-specific genetic programs for proper patterning during craniofacial development (Dollé et al., 1992; Bulfone et al., 1993; Robinson and Mahon, 1994; Depew et al., 2002; Qiu et al., 1997). The *Slc26a1* and *Slc26a2* expression patterns might be part of the jaw-specific gene regulation machinery. Further studies will be required to clarify the mechanisms underlying the regulation of the different expression patterns of *Slc26a1* and *Slc26a2* in the upper tooth germ and lower tooth germ during tooth development.

In *Slc26a2-KO-Δexon2* mice, we observed short, flattened odontoblasts, suggesting the loss of odontoblast polarity (Fig. 3A-D′). Additionally, the expression of *Dspp* and *Dmp1*, well-defined odontoblast differentiation markers, was also decreased in *Slc26a2*-deficient tooth germ compared to control tooth germ *in vivo* (Fig. 4A). These findings highlight the critical role of *Slc26a2* in odontoblast differentiation and dentin formation. Furthermore, *in vitro* experiments showed that odontoblastic differentiation of hDPSCs and mDPCs was substantially suppressed by *Slc26a2* silencing (Fig. 4A and Fig. 5A-D), suggesting that *Slc26a2* directly influences odontoblast differentiation, independent of secondary effects from the surrounding tissues or systemic sulfate insufficiency.

Owing to the postnatal lethality of *Slc26a2*-deficient mice, we evaluated tooth morphogenesis and dentin formation using *ex vivo* transplantation of tooth germ under the kidney capsule, revealing reduced dentin formation in *Slc26a2*-deficient tooth germ compared to control tooth germ (Fig. 4B). *Ex vivo* organ culture of tooth germ is often selected as an alternative method for examining tooth germs from genetically modified mice. Kidney-capsule grafting has previously been reported to provide tooth germs with an *in vivo* biological environment (Ono et al., 2017; Otsu et al., 2012). It is thus possible to retain the physiological features, and morphogenesis of transplanted tooth germs proceeds normally under conditions of kidney-capsule grafting.

GAGs are linear polysaccharides composed of repeating disaccharide units and are typically found as part of proteoglycans (PGs) by attaching to specific serine residues within a core protein. The sulfation of GAGs introduces negative charges at various positions on the PGs, which in turn induces a wide array of biological functions owing to the structural microheterogeneity of these molecules. Heparan sulfate (HS), a highly sulfated GAG, plays a crucial role in both odontogenesis and amelogenesis, as evidenced by previous studies (Bishop et al., 2007; Inubushi et al., 2024). Yasuda et al. (2010) demonstrated the critical role of sulfation in dental tissue development by generating mice deficient in Golgi-associated N-sulfotransferase 1 (NSDT1), an enzyme responsible for the sulfation of HS–PG glycosaminoglycan chains. These *Ndst1* knockout mice exhibited hypodontia in the formation of incisors and molars, alongside abnormal differentiation and organization of odontoblasts (Yasuda et al., 2010). Further supporting the importance of sulfation, Hayano et al. (2012) showed that the removal of sulfate groups from the 6-O position of N-acetylglucosamine by extracellular glucosamine-6-sulfatases SULF1 and SULF2 was significant for odontoblast differentiation and dentin matrix production during dentinogenesis. Specifically, *Sulf1*/*Sulf2* double-null mice displayed a thinner dentin matrix and shorter roots, accompanied by reduced *Dspp* mRNA expression (Hayano et al., 2012). Importantly, HS proteoglycans have also been implicated in the modulation of the canonical Wnt signaling pathway during odontogenesis (Ai et al., 2003; Hayano et al., 2012; Inubushi et al., 2023). Our study's findings of diminished odontoblast differentiation and reduced dentin matrix production in *Slc26a2-KO-Δexon2* mice are consistent with the phenotypes observed in transgenic mice with altered sulfation of HS. Moreover, our RNA-seq data revealed significant downregulation of Wnt signaling pathway-related markers in *Slc26a2*-deficient mDPCs (Fig. 5C,D). This suggests that the attenuation of odontoblast differentiation in these cells may be, at least in part, due to the dysregulation of Wnt signaling during odontogenesis. Given that chondroitin sulfate, another GAG, is also implicated in tooth development (Ida-Yonemochi et al., 2022), it is plausible that *Slc26a2* deficiency affects odontogenesis through the reduced sulfation of GAGs, particularly HS, during tooth development. These insights highlight the critical interplay between *Slc26a2*-dependent sulfate metabolism, GAG sulfation and the canonical Wnt signaling pathway in the proper differentiation of odontoblasts and formation of the dentin matrix.

In conclusion, our study demonstrates that *Slc26a2* is a key sulfate transporter in tooth germ, and its deficiency leads to hypoplasia of the incisors and molars, particularly in the upper jaw. This is the first study to establish the critical role of SLC26A2-mediated sulfate transport in tooth development, providing insights into the mechanisms behind tooth abnormalities in patients with recessively inherited chondrodysplasias caused by *SLC26A2* variants.

## MATERIALS AND METHODS

### Ethics statement

All animal experiments were performed in strict accordance with the guidelines of the Animal Care and Use Committee of the Osaka University Graduate School of Dentistry, Osaka, Japan. The protocol was approved by the Committee on the Ethics of Animal Experiments of Osaka University Graduate School of Dentistry. Welfare guidelines and procedures were performed with the approval of the Osaka University Graduate School of Dentistry Animal Committee (approval number: 3745, 29-033-0).

### Animals

Pronuclear stage embryos from C57BL6/J mice were purchased from ARK Resource (Kumamoto, Japan). Recombinant Cas9 protein, crRNA and tracrRNA were obtained from Integrated DNA Technology. For the generation of *Slc26a2-KO-Δexon2* mice, we used the Technique for Animal Knockout System by Electroporation (TAKE), as previously described (Inubushi et al., 2022). Embryos were washed twice with Opti-MEM solution (Gibco) and aligned in the electrode gap filled with 50 μl Cas9/gRNA (crRNA-tracrRNA complex)/single-stranded oligodeoxynucleotide (200/100/100 ng/μl) mixture. The intact embryos were subjected to electroporation using poring (225 V) and transfer (20 V) pulses. After electroporation, embryos were returned to KSOM Mouse Embryo Media (Millipore Sigma) at 37°C. Genome-edited two-cell embryos were transferred to pseudopregnant ICR female mice oviducts, and genomic DNA from newborn mice was analyzed by PCR. The sequence of gRNA was as follows: left, 5′-AGTCTGAGACCGGTCATGGC-3′; right, 5′-ACAATGAGCTCGACCGGAAT-3′. In all experiments, *Slc26a2^wild/^*^wild^ offspring were used as controls. Genotyping of mice and embryos was performed by PCR with the specific primers listed in Table S1. Similarly to previously reported *Slc26a2* KO mouse models, the *Slc26a2-KO-Δexon2* model utilizes CRISPR/Cas9 technology to target exon 3, with the insertion of a stop codon 137 bases downstream of the start codon. This modification results in a truncated protein that lacks both the transmembrane domain and critical regions necessary for enzymatic activity. Mice were fed *ad libitum* on solid feed and sterile water irradiated with ultraviolet light. The environmental conditions of the animal facility were maintained at constant temperature and humidity and kept under a 12 h light–dark cycle (08:00-20:00 as the light period).

### Tissue preparation, histology and *in situ* hybridization

Maxillary and mandibular tissues from control and *Slc26a2-KO-Δexon2* mice at P0 were collected and fixed in 4% paraformaldehyde. A mild decalcifier, Osteosoft (Sigma-Aldrich), was used for tooth decalcification. Sagittal sections of paraffin-embedded mandible were prepared and used for Hematoxylin and Eosin (H&E) staining and *in situ* hybridization, as previously described (Sarper et al., 2018). The digoxigenin-labeled RNA probes used in this study were prepared using a DIG RNA Labeling Kit (Roche), according to the manufacturer's protocol, using each cDNA clone as the template. The probes were synthesized from fragments of *Slc26a2* (Allen Institute for Brain Science) and were amplified with T7 and SP6 adaptor primers through PCR. After hybridization, the expression patterns for each mRNA were detected and visualized according to their immunoreactivity with anti-digoxigenin alkaline phosphatase-conjugated Fab fragments (Roche). A minimum of three embryos of each specimen type were examined per probe.

### Immunohistochemistry and TUNEL staining

Frozen sections of 12 µm thickness were used for immunostaining and incubated with M.O.M (Mouse on Mouse) Blocking Reagent (Vector Laboratories), 5% goat serum/PBS and 0.1% sodium citrate buffer. Immunofluorescence staining was performed overnight at 4°C on 15 μm sections using polyclonal rabbit anti-Ki67 (anti-MKI67; 1:400; ab15580, Abcam). Sections were then counterstained with 4′,6-diamidino-2-phenylindole (DAPI; 1:500; Dojindo) and mounted using fluorescent mounting medium (Dako). At least three embryos were used for each genotype for each analysis. Apoptotic cells were identified using an *in situ* cell death detection kit (11684795910, Roche) according to the manufacturer's instructions.

### Laser microdissection

We performed laser microdissection as previously reported (Sarper et al., 2018). Dissected heads were freshly mounted in Tissue-Tek O.C.T. Compound (Sakura Finetek, Japan) and immediately frozen. The tissue was then sectioned serially at a thickness of 20 µm using a cryostat (CM 1950, Leica). Sections were mounted on film-coated slides; whole sections were obtained consecutively from the anterior palate at E18.5 and stained with H&E. Odontoblasts from maxillary and mandibular molar were collected in tubes and separated from the sample sections with a manual laser capture microdissection system (LMD6500, Leica). Tissues were serially sectioned at −20°C with a thickness of 25 μm using a cryostat (CM 1950, Leica).

### Gene expression analysis

The protocol for RNA extraction and quantitative PCR (qPCR) analysis was as reported previously (Inubushi et al., 2012). Total RNA was extracted from dissected tissue using an RNeasy Mini Kit (Qiagen), following the manufacturer's protocol, with purity and quantity assessed by a Nanodrop spectrophotometer (Thermo Fisher Scientific). The extracted RNA was reverse transcribed to cDNA using an oligo dt with reverse transcriptase (Takara Bio). For real-time PCR, aliquots of total cDNA were amplified using Fast SYBR Green PCR Master Mix (Applied Biosystems) or Fast TaqMan Fast Universal PCR Master Mix (Applied Biosystems). Data were acquired and analyzed with a Step One Real-Time PCR System using Step One Software, Version 2.1 (Applied Biosystems). The PCR products were quantified using *Gapdh* as the reference gene. The mouse primers used in this study have been previously described (Inubushi et al., 2023). Other primers and probes are listed in Table S2. Each experiment was performed in triplicate.

### RNA-seq

RNA libraries were prepared using a TruSeq RNA Library Preparation Kit according to the manufacturer's protocol. Libraries were amplified by PCR and purified using AMPure XP beads. RNA-seq was performed using a sequencing system (Novaseq 6000, Illumina). The biological significance of differentially expressed genes was explored by volcano plot and GO enrichment analysis using DESeq2.

### Transplantation under the kidney capsule of mice

Control and *Slc26a2*-KO E18.5 upper molar tooth germ were implanted under the renal capsule of 8-week-old BALB/cSlc-nu/nu mice. Four weeks later, the implanted tooth embryos were harvested, and micro-CT was performed. Sagittal slice images (50 µm) were taken with VG Studio and the image processing software The roots were cut in ImageJ, and the volume of the crown dentin was measured.

### Blyscan sulfated GAG assay

A Blyscan sulfated glycosaminoglycan assay kit (Biocolor, Carrickfergus, UK) quantitatively measured sulfated proteoglycans and GAGs in biological samples. The assay was performed according to the manufacturer's instructions. Briefly, 4×10^5^ cells were plated in a T25 flask, and, 48 h later, the cells were treated with 1 μg/ml DS-500 or 50 mM NaClO_3_. Twenty-four hours after treatment, the medium was aspirated, and the cells were rinsed with PBS. The cells were lysed in papain extraction reagent added to the cell monolayer for 3 h at 65°C. Total cell extract containing total GAGs was harvested, and samples were centrifuged at 10,000 ***g*** for 10 min. A total of 100 μl of the supernatant was used for the assay.

### Whole-mount skeletal staining

Mice were fixed in 95% ethanol overnight at room temperature. They were then left in acetone overnight at room temperature and incubated overnight in a cartilage staining solution containing 0.03% (w/v) Alcian Blue, 80% ethanol and 20% acetic acid. The first rinse was performed with several changes of 70% ethanol. To improve visibility of cartilage morphology, washings were terminated before cartilage was completely de-stained. Ossified tissue was stained with an Alizarin Red solution containing 0.005% (w/v) Alizarin Red in 1% (w/v) KOH for 4 h at room temperature. Samples were placed in a 50% glycerol solution containing 1% (w/v) KOH to remove excess staining.

### Micro-CT

Maxilla and mandible were collected from control and *Slc26a2-KO-Δexon2* mice at P0. These tissues were fixed in 4% paraformaldehyde overnight. Embryos were placed in 70% ethanol for 1 day and 100% ethanol for 3 days, then in 100% ethanol with xylene (1:3) for 1 h. The embryos were then placed in 100%, 90%, 80% and 70% ethanol for 30 min each, before being placed in 70% ethanol containing 1% phosphotungstic acid for 1 week for contrast. Both maxilla and mandible were then scanned by micro-CT (R_mCT2, Rigaku) at 90 KV, 200 µA, microfocus 5 µm/voxel size. Volume Graphics (VGstudio) MAX 2.2 software was used for reconstruction of three-dimensional images. Measurements of the maxilla, incisors and molars were performed using VGstudio MAX 2.2 software.

### Reanalysis of public single-cell RNA-seq data

A public single-cell RNA-seq dataset, GSE146855, of mouse incisors was downloaded from the Gene Expression Omnibus database to reanalyze the expression profile in odontoblast differentiation. The data were analyzed using a Seurat (version 4.0.5) package with R studio. A total of 6260 cells were reanalyzed. After normalization, scaling and principal component analysis of the data, cells were clustered using FindNeighbors (dims=1:6) followed by FindClusters (resolution=0.35). The RunUMAP function was used to visualize the cell clusters. Differential expression and cell identification were performed using FindAllMarkers (min.pct=0.25, logFC.threshold=0.25) with the Wilcoxon rank sum test. The top 5 differentially expressed features (cluster biomarkers) and cluster biomarkers are shown in Fig. S1. Visualization of *Slc26a1*, *Slc26a2*, *Slc26a6*, *Slc26a7*, *Slc26a10* and *Slc26a11* gene expression with a dot plot was generated with Seurat function DotPlot.

### Cell culture and lentivirus transduction

hDPSCs isolated from human adult third molars (Lonza) were cultured in Dulbecco's modified Eagle medium (Wako) containing 20% fetal bovine serum (Invitrogen) and 1% penicillin/streptomycin (Sigma-Aldrich), which was designated as growth medium (GM). To induce odontoblast differentiation, hDPSCs were cultured in odontoblast differentiation medium (EM) consisting of alpha Modified Eagle's Medium (Wako) containing 20% fetal bovine serum (Invitrogen) supplemented with 10 nM dexamethasone, 10 mM β-glycerophosphate and 50 μg/ml vitamin C (Sigma-Aldrich). To knock down *SLC26A2* expression in hDPSCs, we used lentivirus-mediated shRNA transduction. Lentivirus particles expressing an shRNA that is validated to deplete human *SLC26A2* (Mission shRNA, TRC Clone ID TRCN8607, MilliporeSigma) and control lentivirus particles expressing an shRNA that does not target any known genes (Mission shRNA, SHC005, MilliporeSigma) were purchased from MilliporeSigma. Lentivirus particles were added to hDPSCs cultured in growth medium supplemented with 5 μg/ml polybrene and cultured for 2 days. Cells transduced with lentiviral shRNAs were selected and maintained in the presence of 10 μg/ml puromycin.

### Alcian Blue technique

Frozen frontal sections of the maxillary from control mice at P0 were stained with Alcian Blue for 30 min and rinsed with tap water for 5 min, followed by two changes of distilled water. The sections were dehydrated with ethanol, washed with xylene and mounted with Dako Fluorescent Mounting Medium (Agilent Technologies).

### Statistical analysis

Statistical methods were not used to predetermine sample size. Statistical analyses were performed with GraphPad Prism 8. Unpaired two-tailed Student's *t*-tests and two-way ANOVA were used under the assumption of normal distribution and observance of similar variance. *P*<0.05 was considered significant. Bonferroni post hoc analysis was performed where applicable. Values are expressed as mean±s.d. For all quantitative experiments performed in this study, statistical analyses were conducted. Variances between groups were similar, and the data were symmetrically distributed. Data shown are representative images; each analysis was performed on at least three mice per genotype. Immunostaining was performed at least in triplicate. For other experiments, the numbers of biological replicates, animals or cells are indicated in the text. No randomization was used to allocate experimental units. There were no exclusion or inclusion criteria; all mutant mice were allocated to each experiment and there were no excluded animals. Confounders were not controlled in the experiments. For the quantitative measurements and statistical analysis, genotype information was masked.

## Supplementary Material

10.1242/dmm.052107_sup1 Supplementary information
