# Supplementary material for: Slc26a2-mediated sulfate metabolism is important in tooth development
Source: Dis Model Mech. 2024 Dec 10;17(12):dmm052107. doi: 10.1242/dmm.052107 (PMC11655027; doi:10.1242/dmm.052107)
Supplement: Supplementary information [file dmm-17-052107-s1.pdf]

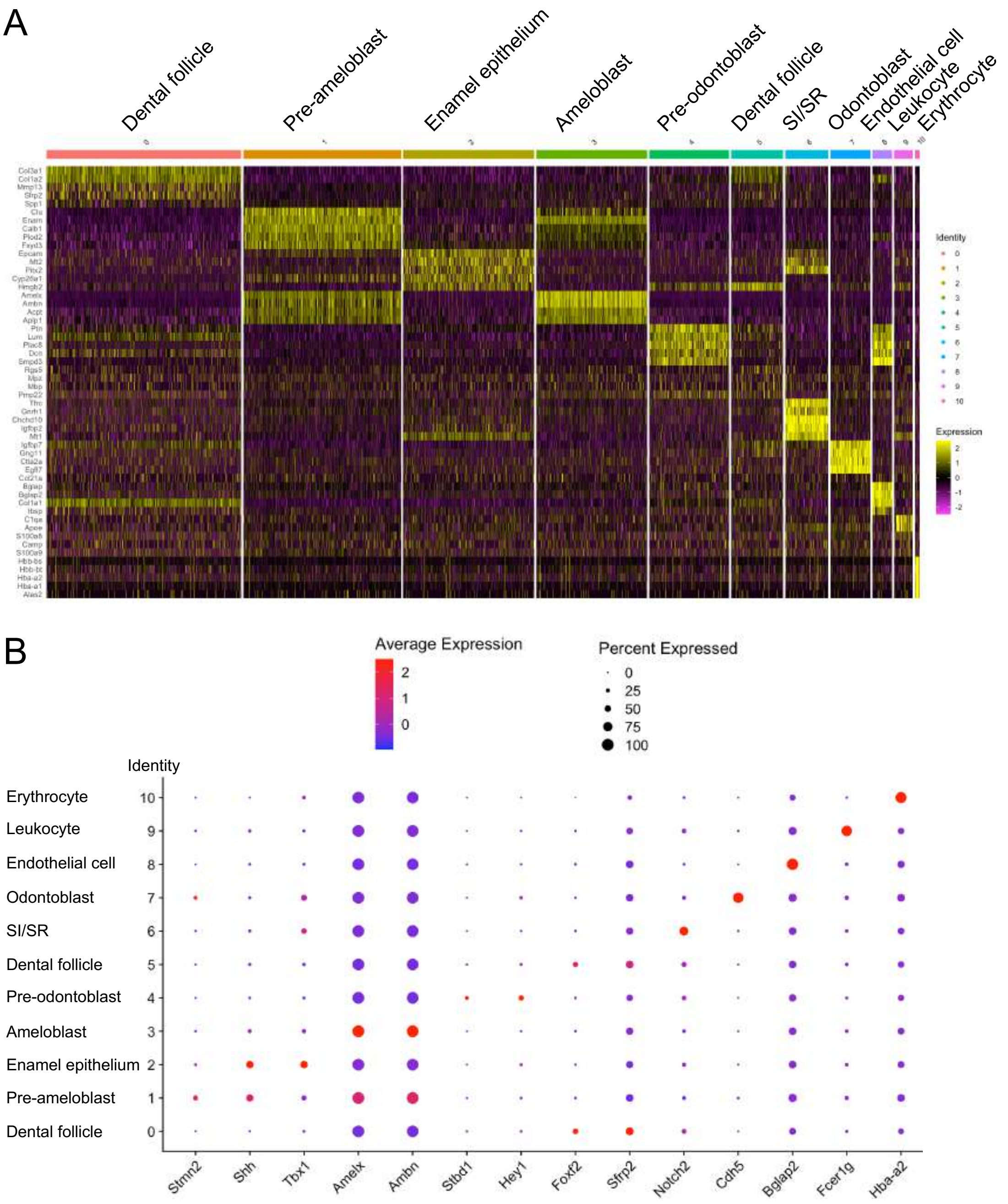

Fig. S1. Reanalysis of the public scRNA-seq dataset (GSE146855) of the isolated mice incisors. (A) Visualization of top 5 differentially expressed features (cluster biomarkers) with Seurat function DimHeatplot. (B) The cluster biomarkers are shown with Seurat function Dotplot.

Disease Models & Mechanisms • Supplementary information

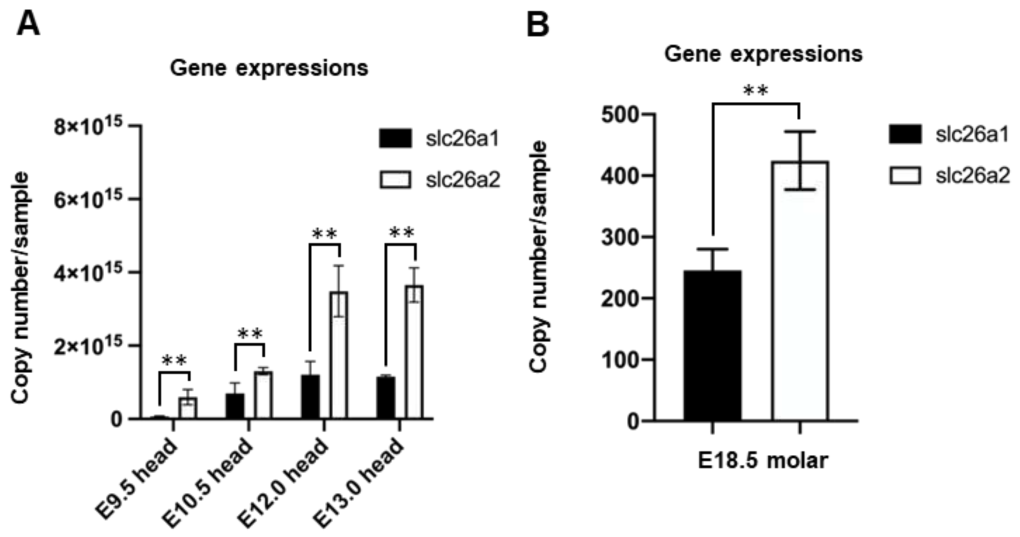

Fig. S2. Expression patterns of *Slc26a1 and Slc26a2* in craniofacial development.

(A) The Expression pattern of *Slc26a1 and Slc26a2* gene during normal development of the craniofacial region. Total RNAs were extracted from the craniofacial region at E9.5-E13.0. At each developmental stage, the expression of *Slc26a2* was found to be markedly higher than that of *Slc26a1*. Furthermore, the expression of *Slc26a2* increased with developmental stage, with craniofacial formation beginning at E9.5 and the tooth bud forming at E13.5 Means ± s.d. (n=3) are shown as horizontal bars. **P<0.01 (two-way ANOVA). (B) The expression pattern of *Slc26a1* and *Slc26a2* during normal upper molars development at E18.5. The expression of *Slc26a1* and *Slc26a2* was evaluated by qPCR. The expression level of Slc26a2 in upper molars was found to be higher than that of Slc26a1. Gapdh was used as an internal control for normalization. Means ± s.d. (n=3) are shown as horizontal bars. **P<0.01 (Students' two-tailed t-tests).

Disease Models & Mechanisms • Supplementary information

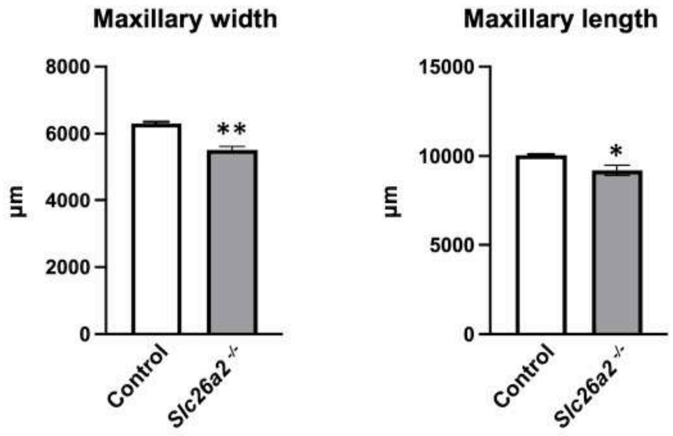

Fig. S3. Measurement of the size of maxilla.

The anterior-posterior length and width of the maxilla were significantly less in *Slc26a2-KO- ⊿exon2* compared to the control mice at E18.5. Means ± s.d. (n=3) are shown as horizontal bars. **P<0.01, *P<0.05 (Students' two-tailed t-tests).

Disease Models & Mechanisms • Supplementary information

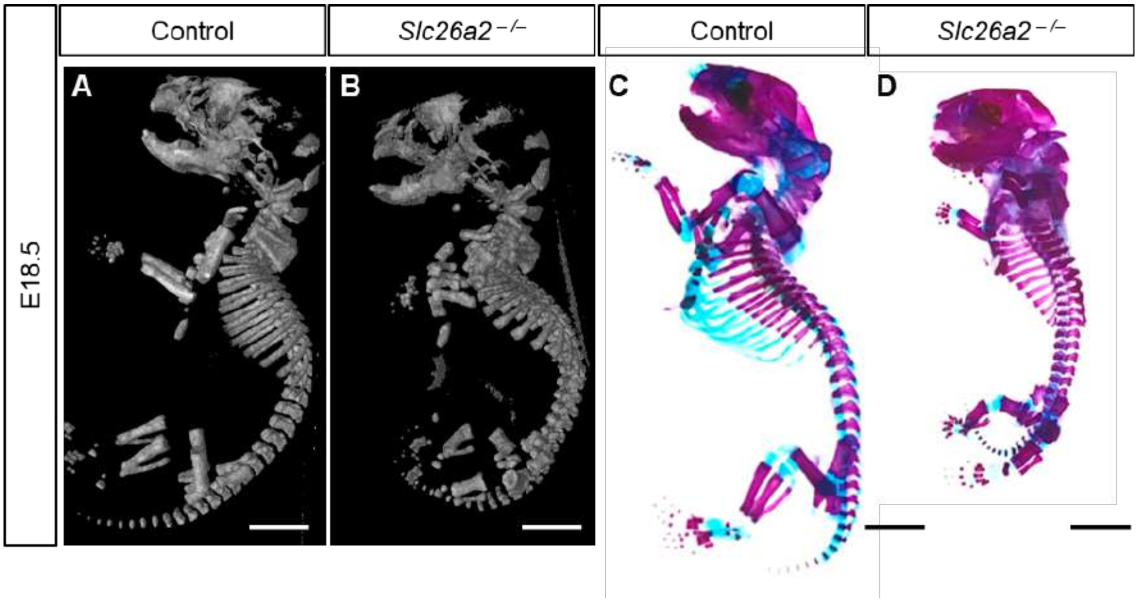

Fig. S4. Morphological analysis of *Slc26a2-KO-Δexon2* at E18.5.

(A) Micro-CT images demonstrate a short stature, small chest, and very short limbs in *Slc26a2- KO-Δexon2* compared to the control mice. The long tubular bone is shorter in length and longer in diameter than the control mice. (B) Whole-mount skeletal preparations showed that *Slc26a2- KO-Δexon2* had chondrodysplasia and reduced alcian blue staining of the cartilage compared to the control mice at E18.5. Scale bars, 4mm. Data shown are representative images; each analysis was performed on at least three mice per genotype.

Disease Models & Mechanisms • Supplementary information

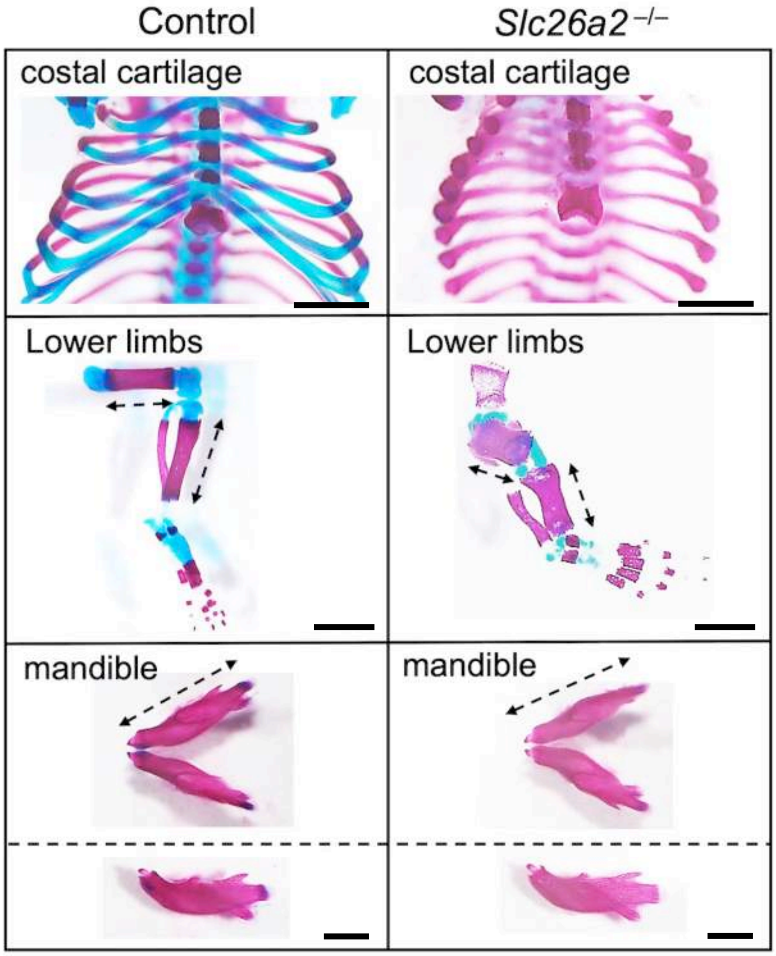

Fig. S5. Whole mount skeletal preparations of *Slc26a2-KO-⊿exon2* at E18.5.
Slc26a2-KO-⊿exon2 showed chondrodysplasia and reduced Alcian blue staining of the cartilage compared to the control mice at E18.5. The mandible size remains consistent with that of control mice. Scale bars, 2mm. Data shown are representative images; each analysis was performed on at least three mice per genotype.

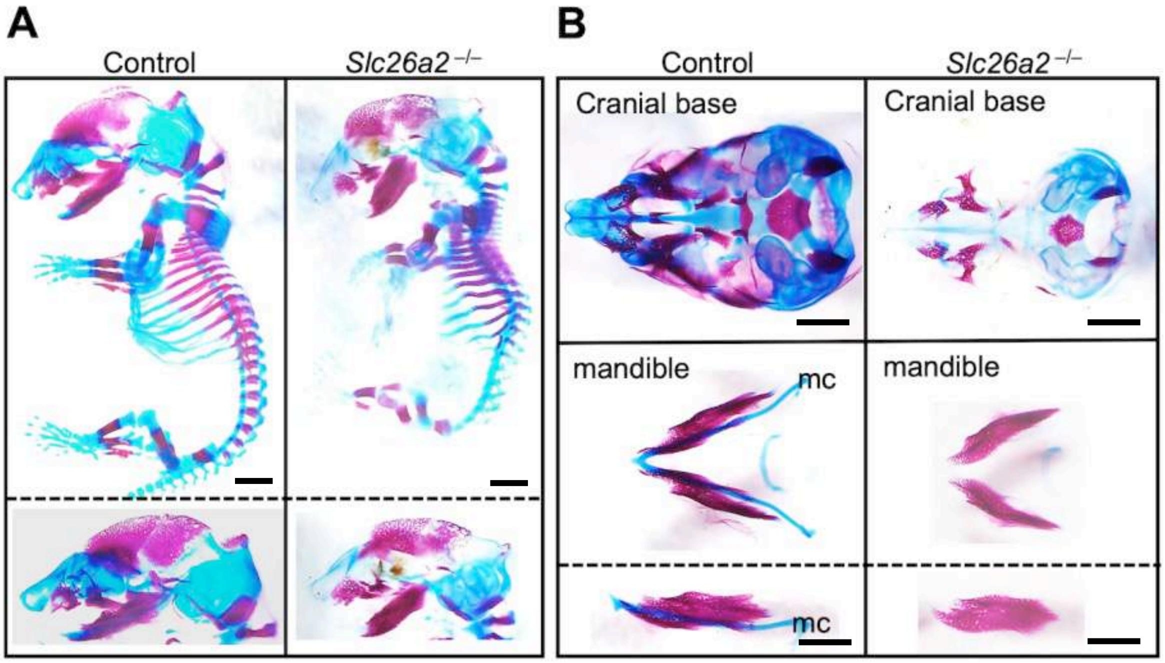

Fig. S6. Whole mount skeletal preparations of *Slc26a2-KO-⊿exon2* at E15.

(A, B) *Slc26a2-KO-⊿exon2* had chondrodysplasia and reduced Alcian blue staining of the cartilage compared to the control mice at E15. Meckel's cartilage (mc) showed decreased Alcian blue staining intensity and hypoplasia Scale bars, 2mm. Data shown are representative images; each analysis was performed on at least three mice per genotype.

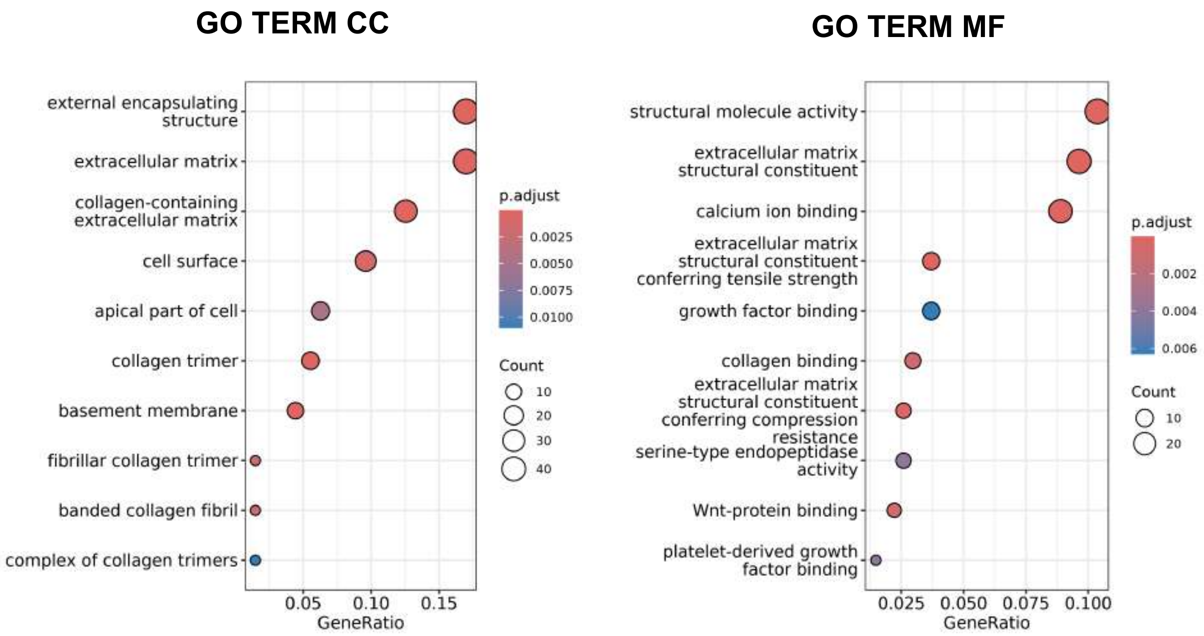

Fig. S7. RNA-seq analysis of genes exhibiting differential expression in Slc26a2 knockdown primary mouse dental papilla mesenchymal cells.

Gene values with |log2FC| > 1 were considered differentially expressed genes (DEGs). Gene ontology (GO) enrichment analysis of DEGs were performed. CC: Cellular Component; MF: Molecular Function.

Disease Models & Mechanisms • Supplementary information

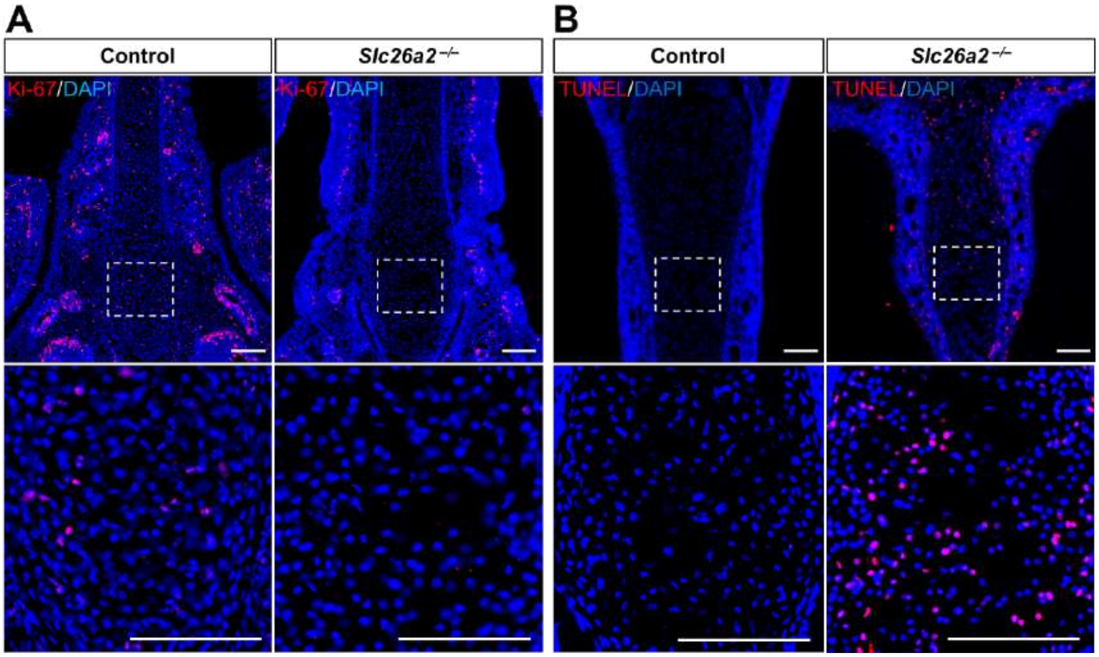

Fig. S8. Deficiency of *Slc26a2* in nasal septal cartilage chondrocytes decreased cell proliferation and increased apoptosis.

(A) Frontal sections of maxillary from control and *Slc26a2-KO-Δexon2* embryos were immuno- labelled with anti-Ki67 antibody. Analysis of cell proliferation in nasal septal cartilage chondrocytes of E18.5 embryos. The number of Ki67 positive cells were decreased in *Slc26a2- KO-Δexon2* compared to the control embryos. The lower panels are the enlarged images of the boxed area in the upper panels. (B) Analysis of apoptosis in nasal septal cartilage chondrocytes of E18.5 embryos. Apoptotic cells were detected by TUNEL assay. The number of TUNEL positive cells were decreased in *Slc26a2-KO-Δexon2* compared to the control embryos. The lower panels are the enlarged images of the boxed area in the upper panels. Scale bars, 50 µm in A-B. Data shown are representative images; each analysis was performed on at least three mice per genotype.

Disease Models & Mechanisms • Supplementary information

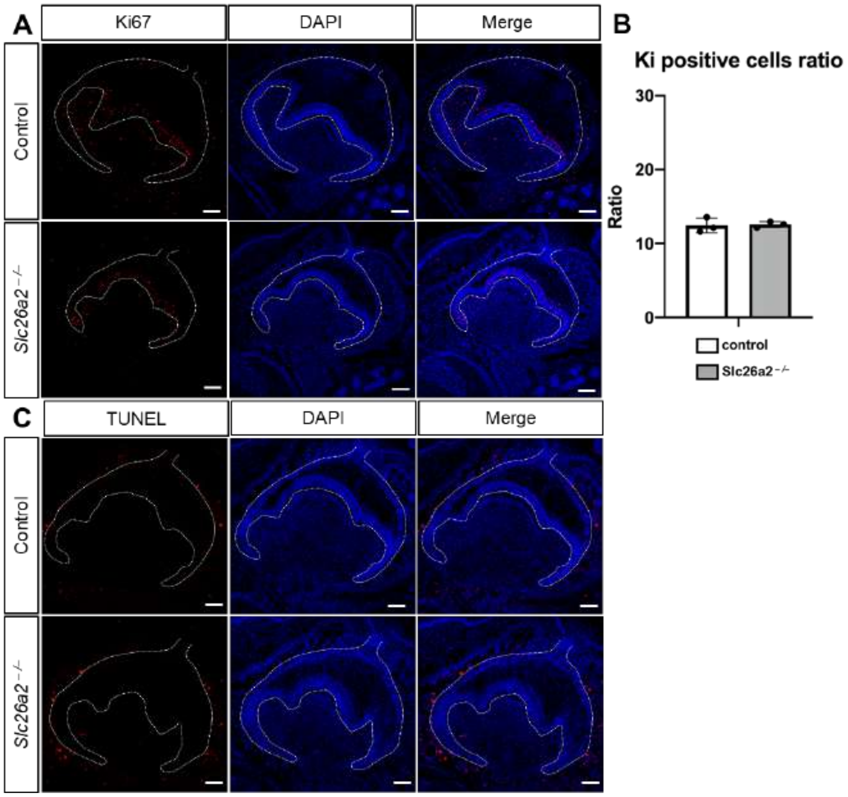

Fig. S9. The cell proliferation and apoptosis were not altered in the tooth germ of *Slc26a2- KO-△exon2* compared to the control mice.

(A) Analysis of cell proliferation in the tooth germ in *Slc26a2-KO-△exon2* and control embryos at E18.5. Frontal sections of the upper tooth germ were immuno-labeled with anti-Ki67 antibody. (B) Quantitative analysis of the number of Ki67 positive cells in (A). Means ± s.d. (n=3) are shown as horizontal bars. *P* values were determined by unpaired Student's *t*-test. There is no statistical difference in the number of Ki67 positive cells between *Slc26a2-KO-△exon2* and control embryos. (C) Analysis of apoptosis in the tooth germ of E18.5 embryos. Frontal sections of the tooth germ of E18.5 embryos were analyzed by TUNEL assays. TUNEL positive cells in dental epithelium and mesenchyme were not detected in both *Slc26a2-KO-△exon2* and control embryos. Scale bars, 50 µm. Data shown are representative images; each analysis was performed on at least three mice per genotype.

Disease Models & Mechanisms • Supplementary information

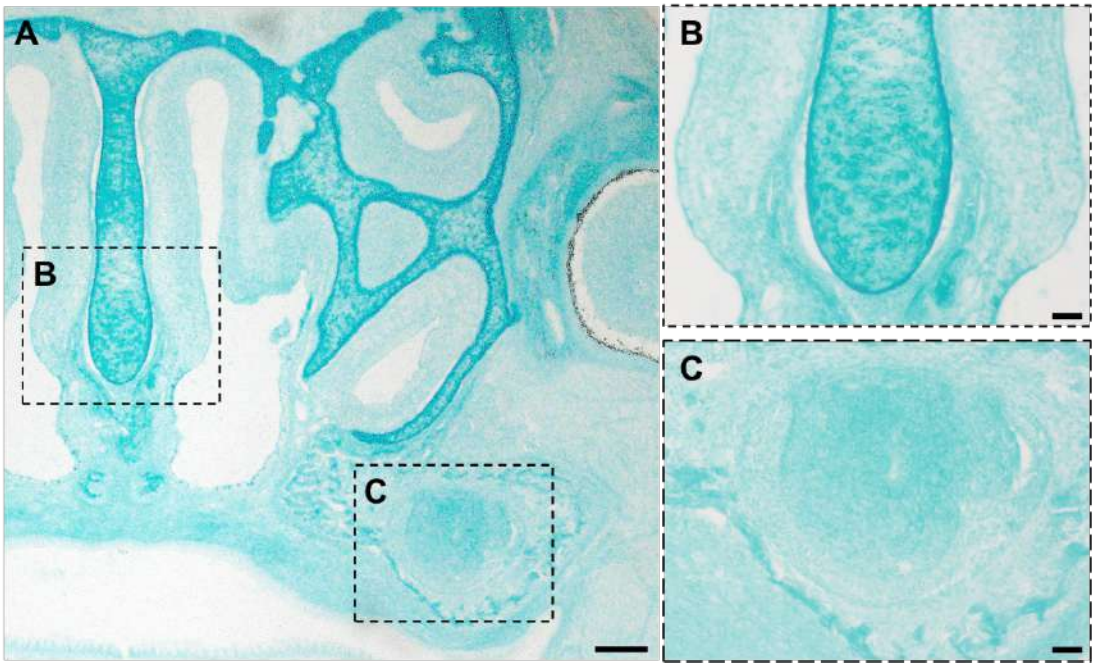

Fig. S10. The sulfation level is extremely higher in cartilage compared to the developing tooth germ. (A) Frontal sections of the maxillary including the nasal cartilage (B) and the upper molar (C) in wild type mice were stained with alcian blue dye at pH2.5. Acidic polysaccharides mainly composed of sulfated glycosaminoglycan were colored with blue. Right panels are enlarged images of the boxed area. The intensity of the alcian blue is higher in the nasal cartilage compared to the developing tooth germ. Scale bars, 100μm in A; 50μm in B, C. Data shown are representative images; each analysis was performed on at least three mice per genotype.

Table S1. Primer sequences for PCR genotyping of mice

Slc26a2 mutant forward	5’-AAGCCTTTGGTTTCCCATCTGA-3’
Slc26a2 mutant reverse	5’-TGGGAATGTGTCCAGCTTAATCG-3’
Slc26a2 wild forward	5’-TTGAGGGCCATCATTTTAGCAGC-3’
Slc26a2 wild reverse	5’-CCAGCTATTCTTCCCCTTCCTCTC-3’

Table S2. Primers used for qRT-PCR

SLC26A2- forward	5′ -CCA GAT GTG GAG GAT TAG CAG AAT GG-3′
SLC26A2- reverse	5′ -ACA GCT TCA TAA TCT CTG CGA ACT TCT TTC AGT GT-3′
DSPP-forward	5’-TGCATTTGGGCAGTAGCAT-3’
DSPP- reverse	5’-TGTCTCTCCAGTGGTTTGCTT-3’
DMP1-forward	5’-CCCTTGGAGAGCAGTGAGTC-3’
DMP1- reverse	5’-CTCCTTTTCCTGTGCTCCTG-3’
Human GAPDH-forward	5’-ACACCCACTCCTCCACCTTTG-3’
Human GAPDH-reverse	5’-TCCACCACCCTGTTGCTGTAG-3’
